# Analysis of bone transport for ankle arthrodesis as a limb salvage procedure for the treatment of septic pilon fracture nonunion

**DOI:** 10.1038/s41598-021-04187-7

**Published:** 2021-12-28

**Authors:** Thomas Rosteius, Sebastian Lotzien, Matthias Königshausen, Valentin Rausch, Charlotte Cibura, Björn Behr, Markus Lehnhardt, Thomas Armin Schildhauer, Jan Geßmann

**Affiliations:** 1 Department of General and Trauma Surgery, BG University Hospital Bergmannsheil, Bürkle-de-la-Camp Platz 1, 44789 Bochum, Germany; 2 Department of Plastic Surgery, BG-University Hospital Bergmannsheil, Buerkle-de-la-Camp-Platz 1, 44789 Bochum, Germany

**Keywords:** Outcomes research, Trauma

## Abstract

Septic nonunion of the pilon region with ankle joint infection is challenging for orthopedic surgeons to treat and is associated with a high risk of limb loss. Therefore, the aim of this study was to evaluate the effectiveness of bone transport for ankle arthrodesis in salvaging the limp after septic ankle destruction of the pilon region. We conducted a single-center, retrospective study including 21 patients treated for septic pilon nonunion with accompanying septic ankle destruction via Ilizarov bone transport between 2004 and 2018. In all cases, the complete excision of the nonunion and the resection of the ankle joint were carried out, followed by treating the bone and joint defect with a bone transport into the ankle arthrodesis. In 12/21 patients an additional flap transfer was required due to an accompanying soft tissue lesion. The overall healing and failure rate, final alignment and complications were recorded by the patients’ medical files. The bone-related and functional results were evaluated according to the Association for the Study and Application of Methods of Ilizarov (ASAMI) scoring system and a modified American Orthopedic Foot and Ankle Society (AOFAS) scale. After a mean follow-up of 30.9 ± 15.7 months (range 12–63 months), complete bone and soft tissue healing occurred in 18/21 patients (85.7%). The patients had excellent (5), good (7), fair (4), and poor (3) results based on the ASAMI functional score. Regarding bone stock, 6 patients had excellent, 7 good, and 6 fair results. The modified AOFAS score reached 60.6 ± 18 points (range, 29–86). In total, 33 minor complications and 28 major complications occurred during the study period. In 2 cases, a proximal lower leg amputation was performed due to a persistent infection and free flap necrosis with a large soft tissue defect, whereas in one case, persistent nonunion on the docking side was treated with a carbon orthosis because the patient refused to undergo an additional surgery. Bone transport for ankle arthrodesis offers the possibility of limb salvage after septic ankle destruction of the pilon region, with acceptable bony and functional results. However, a high number of complications and surgical revisions are associated with the treatment of this severe complication after pilon fracture.

## Introduction

The development of staged treatment protocols, modifications of surgical approaches and modern implants have reduced the overall complication rate in the treatment of pilon fractures. However, in part due to the amount of the initial bone and extent of soft tissue injury as well as the experience of the surgeon, severe complications such as non- and malunion, bone infection and deep soft tissue infection are still associated frequently^1^. Among these complications, chronic osteomyelitis (COM) and septic nonunion of the distal tibia are the most devastating^2,3^. An accompanying ankle joint infection particularly aggravates this already complex situation, since it leads to a combined bone defect of the distal tibia with associated ankle joint loss after resection of the nonunion and ankle joint in order to treat the present infection. Large soft tissue defects may further complicate these conditions with unpredictable outcomes. Despite complex limb salvaging techniques^4^ and many surgical revisions high rates of complications and treatment failure still occur, thus at the end amputation is often required^2^. Therefore, orthopedic surgeons are faced with the question of whether an attempt to preserve the extremity in these situations is still sensible and worthwhile.

A rarely-described limb salvage procedure for these situations of septic nonunion of the distal tibia with accompanying active ankle infection is a bone transport for ankle arthrodesis. This treatment strategy offers the possibility of the simultaneous restoration of the leg length while achieving an anatomically aligned ankle arthrodesis. Therefore, the purpose of this study was to review our results of a staged treatment strategy with bone transport ankle fusion using an external circular frame, with a particular focus on the healing rate, complications and clinical outcomes. To our knowledge, this is the largest case series to date.

## Methods

### Study design

Patients with septic nonunion of the distal tibia and concomitant ankle joint infection after undergoing surgery for a pilon fracture between 2004 and 2018 were retrospectively reviewed. The inclusion criteria were the use of an Ilizarov ring fixator with either classic bone transport for ankle arthrodesis or modified bone transport using an intramedullary cable transportation system (CTS). Patients who underwent primary amputation or patients with incomplete postsurgical follow-up data were excluded. In total, 21 patients fulfilled the inclusion criteria and were considered for further analysis.

The demographic and clinical data, including the age and sex of the patient, medical comorbidities including smoking, active inflammation with detected pathogens, and number of prior operations, were gathered via the medical records at the authors’ institution. The presence of an active infection was either confirmed by microbial detection in tissue and joint samples, or in the case of negative microbial detection, by the presence of a soft tissue defect with an exposed plate and existing joint sinus. The complications were recorded and classified as minor (treated nonoperatively) or major (treated operatively) according to the method used by Katsenis et al.^5^. The pilon nonunion was classified using the Non- union Scoring System (NUSS)^6^. The outcomes were evaluated using the Association for the Study and Application of Methods of Ilizarov (ASAMI) scoring system for bone and functional results reported by Paley^7^ and a modified American Orthopedic Foot and Ankle Society (AOFAS) ankle/hindfoot scale^8^ with a maximum score of 86 points. This modified scale excluding the 14 items on ankle motion has been used in other studies on ankle arthrodesis^3,9^. Total bone loss was calculated as the length of the bone defect after bone resection using an estimated digital measurement tool (Impax, Agfa, Germany). The radiographic consolidation for the distraction gap was measured according to the criteria of Fischgrund et al.^10^ and Paley et al.^11^ using computed tomographies. Overall success was defined as successful limb salvage with complete soft tissue and bone healing without evidence of persistent infection. Amputation, nonunion of the bone or persistent infection was defined as treatment failure.

### Treatment procedure

**Figure 1:**
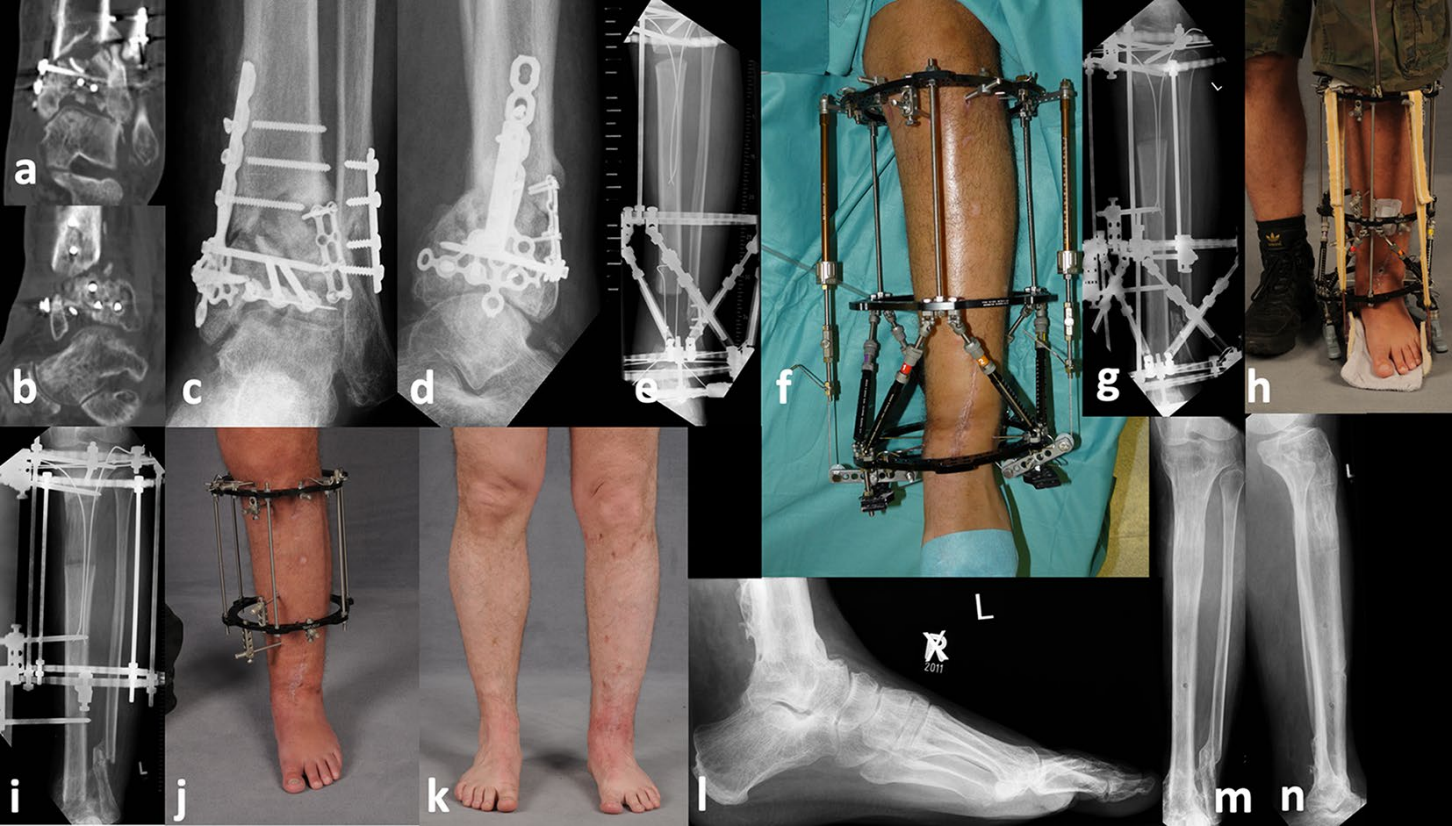
A 40 years old male patient became injured in a motorcycle accident and underwent internal plating of the pilon and distal fibula fracture. Ten months later, the patient was admitted to our hospital with an existing varus and antecurvature deformity of the pilon in combination with a septic nonunion and destruction of the ankle joint (**a**–**d**). Therefore, bone resection was performed with accompanying positioning of a modified Ilizarov fixator with both a cable transportation system (CTS) and hexapod struts for hingefood correction (**e**,**f**). After finishing bone transport and hingefood correction (**g**), the patient was encouraged to full weight-bearing using a rocker food, which was installed at the fixator (**h**). After complete bone healing at docking site, the hexapod hingefood fixator was removed (**i**) to allow free full weight-bearing and stimulate the consolidation of the regenerate (**i**,**j**). After 9 months and complete healing of the regenerate the fixator could be removed. Picture (**k**–**n)** demonstrate the final result, showing total bone and soft tissue healing with optimum axial alignment at 2 years after removal of the fixator.

**Figure 2:**
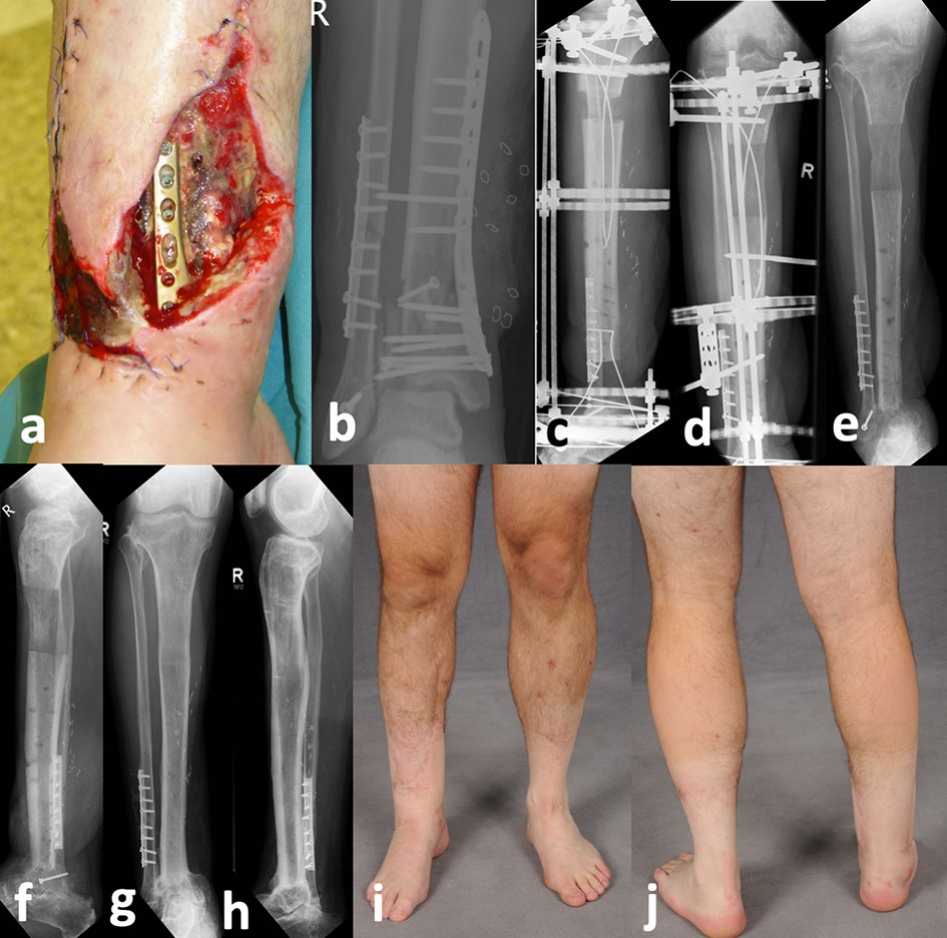
A male patient who was 37 years old fell as a roofer from a height of 5 m resulting in a 3° open lower leg fracture. After three previous operations with internal plating and two debridement’s, the patient was admitted to our hospital with a septic, exposed distal tibia and a detection of Serratia marcescens on the plate and of the ankle joint (**a**,**b**). In the first step, bone and necrotic soft tissue resection and positioning of the flexible cable was performed. The plastic surgeon used a free ALT flap transfer to close the soft tissue lesion. After healing of the soft tissue, the OTA fixator was removed, and the ring fixator with a CTS was applied (**c**). Following 4 months of transport, the docking operation was performed (**d**). Subsequently, the ring fixator was removed after 7 months of consolidation (**e**,**f**). At the final follow-up, at 4 years after bone transport, complete bone and soft tissue healing with full weight-bearing had been reached (**g**–**j**).

Radical debridement with resection of all infected and necrotic bone until the point of cortical bleeding as well as resection of all infected soft tissue was performed in all cases at the beginning of treatment. Three to 5 tissue samples were obtained for the microbial examination. The incubation was ensured for 14 days. According to the antibiogram, the infection was treated with antibiotics for 6 weeks. In cases with a remaining deep soft tissue defect and without the possibility of wound closure, a monolateral OTA frame was assembled to allow free soft tissue transfer for the next days. Following healing of the free flap and soft tissue, the OTA frame was changed to the definitive ring frame, and proximal osteotomy was performed for bone transport. In cases that did not need free soft tissue transfer, the ring fixator was assembled within the first operation, and a tibial osteotomy was performed. In all patients, a four-ring circular fixator was used for bone transport. The frame was individualized for each patient with respect to the ring size. In 14/21 patients, an intramedullary CTS was used for distraction osteogenesis (Figs. 1, 2), whereas in 7 patients a classic bone transport was utilized. The intramedullary cable (Ilizarov cable, 1.5*1200 mm, Smith & Nephew, Inc., Memphis, USA) for bone transport in the CTS group was positioned when segmental tibial bone resection and soft tissue debridement was performed. It was inserted through the proximal bone end of the tibia (Figs. 1, 2)^12^, prior to a possibly required free flap transfer. Transport was started 7–10 days after the osteotomy at a rate of 1 mm/day and 4 evenly distributed steps of 0.25 mm per day. The patients performed domestic transport themselves, and radiological progress monitoring of the transport was performed every 2 weeks. At the time the bone transport segment was docked on the talus, iliac crest bone grafting was performed in all patients. In the cable transport cases (14 patients), the cable was removed during the docking operation, and additional wires and half pins were inserted in the transport bone segment. An osteotomy of the fibula was also performed to allow regular compression of the talotibial arthrodesis over the following weeks. After the docking operation, the patients were encouraged to bear full weight. Clinical and radiological follow-ups were scheduled every 4 weeks. The frames were removed in the outpatient clinic after bony consolidation was observed on normal X-ray or CT scans. If bony union at the site of arthrodesis was achieved prior to bony consolidation of the proximal callus regenerate after distraction, the distal rings were removed to enable the patient to walk with greater ease (Fig. 1j). In cases of delayed consolidation of the regenerate area, the Ilizarov fixator was removed, and internal plating was performed to reduce the external fixation time and provide sufficient stability.

### Statistical analysis

The continuous variables are described using means, standard deviations, and ranges. The categorical variables are expressed as absolute frequencies and percentages.

### Ethical statement

There is a positive statement of the Institutional Review Board for this work (reg-nr. 4554-12).

### Informed consent

Informed consent was obtained from all individual participants included in the study.

## Results

### Demographics

In total, six patients were female (28.6%), and the mean age of all the patients was 48.4 ± 11.6 years (range 30–72). The mean follow-up was 30.9 ± 15.7 months (range 12–63 months). The mean bone defect size was 7.4 ± 2.3 cm (range 3.4–11.0 cm). In 10 (47.6%) patients, vascularized free flap transfer was necessary, and in 2 patients, local flap transfer was necessary prior to the beginning of bone transport due to existing major soft tissue lesions at the time of the initial visit at our hospital. Table 1 shows the characteristics of the study group.

**Table 1:** Patient characteristics.

Clinical data	
Age (years)	48.4 ± 11.6 (range, 30–72)
Sex	
Male	15 (71.4%)
Female	6 (28.6%)
**ASA score**	
1	3 (14.3%)
2	14 (66.7%)
3	4 (19.0%)
NUSS score	61.3 ± 6.7 (range 48–72)
BMI (kg/m^2^)	28.4 ± 4.7
Drug abuse	2 (9.5%)
Smokers	13 (61.9%)
**Initial OTA fracture type**	
43 B2	1 (4.8%)
43 C1	4 (19.0%)
43 C2	4 (19.0%)
43 C3	12 (57.1%)
Soft tissue lesion	12 (57.1%)
No. of previous operations	3 ± 1.2 (range 2–7)
**Pathogens**	
*Staph. aureus*	4 (19.0%)
*Staph. epidermidis*	4 (19.0%)
*Serratia marcescens*	1 (4.8%)
*Strept. agalactiae*	2 (9.5%)
*E. coli*	1 (4.8%)
*Propioni. acnes*	1 (4.8%)
*Enterococcus*	3 (14.3%)
*Bacillus *spp.	1 (4.8%)
*MRSA*	1 (4.8%)

### Bone-related and functional results

The overall success rate with limb salvage and complete bone and soft tissue healing after septic ankle destruction for pilon fractures using Ilizarov bone transport was 18/21 (85.7%). The mean external fixation time was 335 ± 103 days (range 161–532 days), and the average distraction time was 120 ± 70 days (range 40–354 days). According to the ASAMI functional score, 5 patients had excellent, 7 good, 4 fair, and 3 patients poor results, respectively. Regarding the bone results, 6 patients had excellent results, and 7 patients had good results; 6 patients had only fair results. Fourteen patients had a mean residual bone shortening of the tibia of 2.2 ± 1.1 cm. In 4 patients a residual mean varus malalignment of 10.3° ± 2.9° was registered, whereas in 4 patients a residual tibial valgus of 8.5° ± 2.9° remained. Three patients had a mean residual tibial antecurvation of 13.3° ± 6.8°, one patient had an ankle fusion with 12°plantar flexion. The modified AOFAS score reached 60.6 ± 18 points (range 29–86). In 9 patients with delayed consolidation of the regenerated area, the Ilizarov fixator was removed, and internal plating was performed. Table 2 shows the consolidation and bone healing results.

**Table 2:** Consolidation and healing of the bone regenerate.

	Consolidation (days)	Distraction (days)	Healing index (day/cm)	Distraction index (day/cm)	Consolidation index (day/cm)	Distraction–consolidation index (day/cm)
Mean	407.3	119.9	47.0	15.3	60.6	46.8
Standard deviation	321.1	70.2	14.8	5.1	46.4	14.8
Minimum	103	40	29.7	9.8	20.2	29.7
Maximum	1230	354	85.7	30.4	161.4	85.7

Consolidation: time between docking and removal of the fixator/days.

Distraction: time between starting bone transport and docking.

Healing index: wearing time fixator (days)/defect size.

Distraction index: distraction time/ defect size.

Consolidation index: consolidation time/defect size.

Distraction-consolidation index: time between starting bone transport and removal of the fixator/ defect size.

### Complication and failure rates

In total, 28 major complications led to revision surgery (median 1 complication/patient, range 0–3 complications/patient). Thirty-three minor complications, which occurred during the study period (median 1 complication/patient, range 0–4 complications/patient), could be treated non-surgically. Tables 3 and 4 show the frequencies of the major complications with the corresponding treatment.

**Table 3:** Major complications with corresponding treatment.

Major complications during distraction	Total
Cable breakage of the CTS with the necessity of renewed attachement of the flexible cable	3
Incorrect use of the click system of the telescopic rods of the CTS with non-movement of the transport segment and necessity of renewed osteotomy	1
Axial misdirected transport of the tibia during classic bone transport with need of revision of the fixator and the transport segment	2
Free flap necrosis with renewed flap transfer	2
Persistent Deep soft tissue infection with revision surgery	3
Cut out of cable olive wires with need of repositioning	2
Unguis incarnatus with need of Emmert Plastic due to lack of feed care with attached fixator	1
Total	14

**Table 4:** Major complications with corresponding treatment.

Major complications during consolidation	Total
Pin loosening with renewed pin positioning	1
Nonunion at docking site with renewed bone grafting	4
Fracture of the regenerate with internal plating	2
Insufficiency of the regenerate with internal plating	7
Total	14

The failure rate for the treatment of septic ankle destruction using Ilizarov bone transport was 3/21 (14.3%). In 2 cases, a proximal lower leg amputation was performed, and in 1 case, persistent nonunion at the docking site was treated with a carbon orthosis because the patient refused to undergo another surgery.

Table 5 shows the reasons for failure and additional treatment.

**Table 5:** Failures and further treatment.

Patient no.	Reason to failure	Further treatment
8	Persistent nonunion at docking side with patients’s refusal to undertake further surgical intervention	Prescription of an orthosis with stabilization of the nonunion
13	Major soft tissue lesion with free flap nekrosis and without the possibility of re-transplantation	Proximal lower leg amputation
15	Persistent osteomyelitis, soft tissue infection with regenerate infection	Proximal lower leg amputation

## Discussion

In our study, we demonstrated that bone transport for ankle arthrodesis in cases of septic pilon nonunion with ankle joint infection is a suitable salvage procedure to preserve the affected extremity. Despite profound bone loss with a mean defect size of 7 cm and an accompanying high rate of large soft tissue defects with the necessity of free flap transfer, it was possible to achieve bone union, restore the leg length, and achieve acceptable bone and functional results without infection, although various complications occurred during this complex treatment.

In general, the ankle joint should be preserved if there is no joint destruction or chronic infection. If the infection cannot be controlled initially and progresses to chronic osteomyelitis with ankle joint infection, reconstructive surgery with limb salvage for the acute and chronic infection in the distal end of the tibia and ankle joint should be performed, but this surgery remains very challenging, despite all the surgical progress made over the last decades^9,13^. Eradication of the infection, reconstruction of the bone defects, solid union of the arthrodesis, correction of malalignment and soft tissue closure need to be accomplished to restore limb function. In these complex situations, the surgeon is faced with the question of whether limb salvage is still feasible and whether amputation is the more realistic and practical treatment option^2,5^. However, if solid union and anatomically aligned arthrodesis are obtained, good functional outcomes can be expected in the long term, even for revision cases^5^.

Therefore, we present a working, staged treatment protocol with the use of an Ilizarov fixator and distraction osteogenesis for ankle arthrodesis. The Ilizarov fixator offers a treatment method with the advantages of external fixation in the existing septic conditions as well as the possibility of early weight-bearing after the transport has been completed. Moreover, it offers a high degree of flexibility for these heterogeneous cases due to the possibility of the following optional technical modifications:

For example, the use of an intramedullary CTS offers different advantages, especially in cases with a combined severe bone loss and large soft tissue defect in the pilon region^12^. The use of an intramedullary CTS promotes soft tissue healing and especially free flap healing due to the absence of external soft tissue cutting rods, pins or cables, which are needed for the classic transport method^12^. Second, intramedullary CTS allows targeted transport with axially aligned regenerated bone, which is particularly important in bone transport for ankle arthrodesis since the tibia has a small diameter and needs to be moved into the right position with respect to the talus, which becomes more difficult as the defect size increases. This is supported by the fact, that axial misdirected transport only occurred in the classic transport group (Table 3). At first glance, the additional docking operation that is required to remove the flexible cable of the CTS seems disadvantageous with respect to classic bone transport. However, since autologous bone grafting at the docking site is the current “gold standard”^14–16^ and delayed bone healing is often observed at the docking site^17^, we performed both cancellous bone grafting at the docking site and removal of the flexible cable. This combined procedure may lead to significantly lower rates of delayed union at the docking site than those reported in the current literature^17^. However, a specific problem of the CTS group that had to be mentioned was a cable breakage that occurred in three cases, necessitating a renewed attachment of the flexible cable.

Furthermore, the flexibility of the Ilizarov ring fixator also enables the retraction of hexapod struts, which can still simplify/ optimize a gradual adjustment of the arthrodesis (Fig. 1).

Generally, proximal osteotomy is problematic in all types of transport, as it can complicate future transtibial amputation in cases with treatment failure and can lead to knee exarticulation due to persistent infection or proximal regeneration insufficiency.

A recent study by Brauns et al. investigated the results of 10 patients who also underwent bone transport ankle arthrodesis due to septic pilon nonunion. However, most problems that the authors reported were related to the consolidation of the docking site, and additional retrograde nailing after removal of the ring fixator was needed in 5 out of 10 patients^17^. Other studies involving distraction osteogenesis have also suggested staged treatment protocols including intramedullary nailing for distal tibial bone defects^13,18,19^. In particular, the integrated technique^19^ and lengthening over a nail^18^ should be mentioned here. However, it should be considered that retrograde nailing destroys the non-affected subtalar joint, leading to additional subtalar joint arthrodesis. Since the flexibility of the hindfoot and midfoot is a compensatory mechanism for minor malposition of the ankle^20^, this procedure needs to be debated. Nevertheless, there is also an additional risk of an implant infection after septic nonunion of the tibia with major soft tissue lesions.

Additional techniques have also been proposed to obtain solid union and address bone defects^4,5,9,17,18,21–24^, but all studies describe high complication rates with long treatment durations, so no standard of treatment exists. For example, acute tibial shortening at the site of arthrodesis followed by gradual lengthening of the proximal tibia is a well-defined and established technique^25,26^. Bone defects that exceed the size in which acute shortening is not possible due to soft tissue and vascular system problems are considered critical. While acute shortening of up to 8 cm is feasible in the proximal tibia, 3–4 cm is the recommended limit for acute shortening in the distal tibia^27^. Excessive shortening may result in soft tissue swelling and necrosis due to arterial kinking and occlusion and venous and lymphatic stasis^27–30^. These large bone defects, as in our study exist (mean defect size 7.4 cm, only 3 patients with a bone defect < 5 cm), can therefore preferably be reconstructed using bone transport ankle arthrodesis, whereby lengthening of the tibia can be performed simultaneously with ankle arthrodesis as a single-stage procedure or in a staged fashion in cases of active infection. The anatomical leg length can be maintained with this approach. Another alternative method is gradual shortening for cases of larger bone defects.

In general, however, all procedures are considered extremely time-consuming and prone to complications. Additional accompanying risk factors, such as large soft tissue lesions, smoking^31^ or limited patient compliance, could complicate the treatment. Nevertheless, to our opinion, limb salvage should be preferred to early amputation since the literature shows equivalent short- and long-term outcomes^32–35^. In addition, there is evidence that limb salvage is psychologically more accepted by patients with severe lower limb trauma than is amputation, although the physical outcome and level of function are better than or the same as those of amputation^36,37^.

This study has several limitations. The type of transport with either CTS or classic transport or the use of technical modifications like hexapod struts was selected based on the clinical parameters of each individual patient and the surgeon’s judgment, which may have introduced bias in this study regarding the complications and treatment duration. Several technique-related limitations also exist. A high level of patient compliance is needed for domestic transport to be performed by the patient. This requirement can lead to an increased occurrence of complications, such as inadequate care of the fixator with an increased incidence of pin infections, transport in the wrong direction, early ossification of the regenerate or pulling out of the cable. In addition, a larger amount of material (transport rods, roles, flexible cable wires) is necessary for CTS than for classic bone transport.

## Conclusion

In cases of septic ankle joint destruction with concomitant nonunion after pilon fractures, leading to critical-sized bone loss and severe soft tissue lesions, a staged, interdisciplinary treatment protocol with the use of Ilizarov bone transport for ankle arthrodesis is a worthwhile limb-sparing procedure. Even though the complexity of these cases leads to high complication rates and long consolidation and external fixation times, the final outcome can be satisfactory.
